# The Effect of Baby Walker on Child Development: A Systematic Review

**Published:** 2017

**Authors:** Shervin BADIHIAN, Negin ADIHIAN, Omid YAGHINI

**Affiliations:** 1 Child Growth and Development Research Center; School of Medicine; Isfahan University of Medical Sciences; Isfahan; Iran.; 2 Department of Medicine; Najafabad Branch; Islamic Azad University; Najafabad, Iran.; 3 Child Growth and Development Research Center, School of Medicine, Isfahan University of Medical Sciences, Hezar Jarib St, Isfahan, Iran.

**Keywords:** Baby walker, Infant walker, Development

## Abstract

**Objective:**

Baby walkers are used all around the world as fun equipment without any dangers. In contrast with public beliefs, some researchers have claimed they can cause developmental delay. We aimed to investigate their effect on child development through a systematic review.

**Materials & Methods:**

We searched PubMed, Google Scholar, EMBASE, and Scopus for related articles in English and included all study designs. All articles, which fulfilled the inclusion criteria, were included without considering the year of publication.

**Results:**

Of 315 articles found in PubMed, 1630 citations in Google Scholar, 18 articles in EMBASE, and 38 papers in Scopus, only 9 articles fulfilled the inclusion criteria. Among them, a cohort study and two cross-sectional studies reported developmental delay in thaspects in baby walker users. Other studies including clinical trials did not show any developmental delay in these children.

**Conclusion:**

Evidence against baby walker is not enough regarding its negative effect on child development. This subject needs to be addressed more, considering a large number of baby walker users worldwide.

## Introduction

Baby walkers are known as fun entertaining equipment used for infants between 4 to 12 months of age globally (1). About 50%-77% of parents of infants 3 to 12 months use baby walker (2, 3). The use of baby walker is based on cultural beliefs and personal interests of parents (4). Despite the popularity of baby walkers, there are notable concerns about them. They are highly associated with accidents and injuries, happening in 12% to 50% of users (5, 6). However, the missing point not neglected is the developmental delay that may occur among walker users (5). 

The possible developmental delay can be discussed from two aspects. First, they provide precocious locomotion in infants, which may interfere with the natural process, that an infant needs to take to develop (7, 8). Second, they prevent visual experience of moving limbs because of their design, believed to have a critical role in development of motor systems (9). For these reasons, especially walker related injuries, baby walker sale has been banned in Canada since 1989 (10) and the American Association of Pediatrics has not recommended baby walker as well (5). 

In this study, we summarize previous findings on the effect of baby walker on child development.

## Materials & Methods

This study was conducted in Apr 2016 and updated in Apr 2017 using databases of Medline, EMBASE, Scopus, and Google Scholar. All articles, which fulfilled the inclusion criteria, were included without considering the year of publication. PubMed query was (((baby OR infant OR pediatric [MeSH Terms] OR pediatric OR child)) AND (walker OR runner)) AND (development [MeSH Terms] OR walk). Google Scholar was searched for papers with the terms (“baby walker” OR “infant walker”) AND (development OR walk) anywhere in the article, without any limitation. EMBASE was also searched with keywords “baby walker” OR “infant walker” AND “development OR walk. Same keywords were used to search Scopus. Reference lists from potentially relevant papers were also hand searched to find any additional studies missed during our search.

Two coauthors reviewed the titles and abstracts of all citations found by literature search and full texts of relevant papers were received thereafter. The inclusion criteria were applied as follows: 1) Studies that had evaluated the effect of baby walker on child development, 2) Published in full manuscript, 3) Published in English. The review included both interventional and observational studies as well as case reports. The interventional studies were evaluated using PEDro scale.

Two reviewers evaluated eligible articles and data were extracted about the first author, year of publication, title, journal, study population, sample size, study design, methods, demographic factors, study outcomes, study findings, and study limitations. The extracted data were entered into sheets to be compared as reported in this paper.

Results

**Fig 1:**
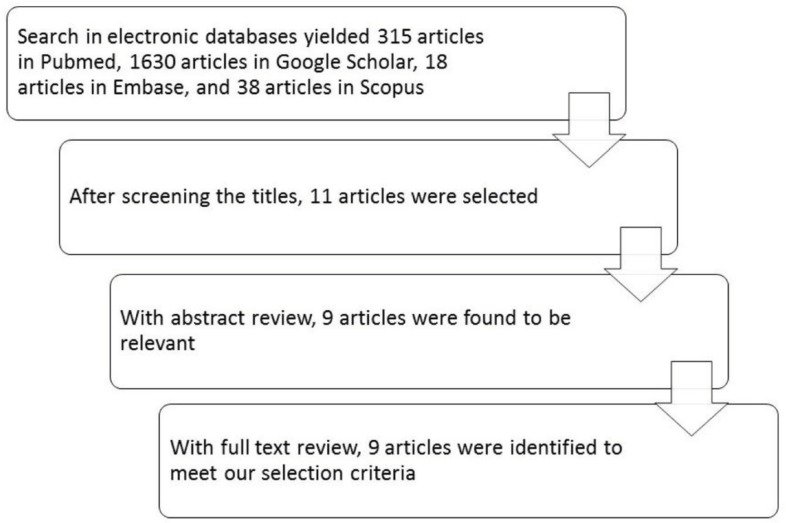
Diagram of the searches for the systematic review of baby walker effects on child development

Totally, 315 articles were found in PubMed, 1630 in Google Scholar, 18 in EMBASE, and 38 in Scopus. Reviewing the titles and abstracts followed by the review of the full manuscripts of relevant articles, led to identification of nine articles that met our selection criteria including two clinical trials, six observational studies, and a case report. A final update of the search was done in Apr 2017 and no new result was added (Figure 1, Table 1).

**Table 1:** Details of extracted data for this review

**Table 1.** Data extraction								
Author /year	Study design	Sample size (No)	Duration of follow-up	Age at the baseline^1^	Frequency of walker use^2^	Outcome measurement/ Intervention	Finding	Problems
Kauffman and Ridenour /1977	Clinical trial	12 (6 pairs of male/female twins)	68 d (until onset of walking)	10 (mean)	2	The mean age of gait acquisition (walking 4 steps independently)/ One child from each pair used baby walker	**No difference** in gait acquisition age between two groups	High mean age of participants at the beginning- No information on sample size calculation-Failure to define the study population clearly- PEDro scale=5
Ridenour /1982	Clinical trial	30 (15 pairs of twins)	~250 d (until onset of walking)	4 (mean)	1	The mean ag of gait acquisition (walking 3 steps independently)/ One child from each pair used baby walker	**No difference** in gait acquisition age between two groups	No information on sample size calculation-Failure to define the study population clearly- PEDro scale=5
Crouchman et al /1986	Cross-sectional	66	-	8-12 (no mean available)	1 in the low-user group (22 patients); 4 in the high-user group (20 patients)	Age at onset of sitting, walking, and prone locomotion	**Delay** in onset of prone locomotion in high-user group- **No difference** between age at onset of sitting or walking among groups	Recall bias-Lack of further evaluations for the children using walker excessively- Small number of cases in each group
Thein et al /1997	Cross-sectional	185	-	7-10 (no mean available)	<1 (29 patients); 1-2 (50 patients); >2 (88 patients)	DDST-S^3^ results (normal, questionable, abnormal)	**12 abnormal results** **and 6 questionable results** among walker users	Recall bias- Absence of control group
Siegel and Burton /1999	Retrospective cohort	109	3 months (until onset of walking)	4.8 (mean age at walker onset)	2.3 (56 walker users)	Age at onset of sitting, crawling and walking- Bayley motor and developmental scores	**Motor developmental delay** regarding sitting, crawling, and walking among walker users- **Lower Bayley scores**	Various ages at onset of using walker- Various frequencies of using walker
Garret et al /2002	Cross-sectional	190	-	26 wk (median)	Not available (102 walker users)	Age at onset of raising the head when prone, sitting with support and alone, crawling, standing with support and alone, walking with support and alone	**Delay** in crawling, standing alone, and walking alone in walker users	Failure to verify study outcome- Failure to randomize the study group- Not reporting the frequency of using baby walker
Chagas et al /2011	Cross-sectional	26	-	Not available	Not available (14 walker users)	Taking five steps without support	**No difference** between two groups regarding gait acquisition	Incomplete reported descriptive data- Not reporting the frequency of using baby walker- Small number of cases in each group
Schopf et al /2015	Cross-sectional	20	-	486.05 d (mean)	Not available (10 walker users)	Age at walking skills development, current motor development measured by AIMS^4^	**Earlier **walking skills in baby walker users, **No difference** in current motor development (using AIMS) between two groups	Incomplete reported descriptive data- Not reporting the frequency of using baby walker- Small number of cases in each group- Qualitative method

The first clinical trial on this issue was conducted that enrolled six pairs of twins, with mean age of 10 months (11). One child from each pair used walker for two hours per day (mean) and they were followed until starting walking four steps independently, and the mean age of gait acquisition was compared between walker users and non-users (11). No statistically significant difference was reported in gait acquisition age between two groups. A major methodological problem in this study was the mean age of participants at the beginning of the study (10 months) which is late considering the age in which families start using walkers. Five years later, this issue was repeated with 15 pairs of twins, with mean age of 4 months, to eliminate limitations of the previous study (12). Subjects were followed that could walk three steps independently, and found no statistically significant difference in gait acquisition age between two groups (12). Both of these studies failed to define the study population clearly and do not present any information about sample size calculation.

Besides, 66 infants were enrolled aged 8-12 months and divided them into three groups of high-user, low-user, and non-user, and interviewed their caregiver to evaluate motor development. The high-user group was showed delay in onset of prone locomotion; however, no difference was seen between age at onset of sitting or walking among groups (13). Overall, 185 infants (167 of them using walker) were studied and evaluated developmental delay using Denver Developmental Screening Tool (DDST-S). Among walker users, 12 (7.2%) had abnormal DDST-S results and six (3.6%) had questionable results while all of walker-non-users had normal results. In addition, among 18 cases with abnormal or questionable results, 17 of them showed gross motor developmental delay and one showed speech and language developmental delay. Using baby walker might cause developmental delay (14). A major limitation of this study was absence of a control group. A retrospective cohort was designed with enrolling 109 infants with mean age of 4.8 months of onset of using walker in the study. Subjects were examined for the age at onset of sitting, crawling, and walking and reported motor developmental delay in walker users compared to non-users in all the mentioned areas as well as lower Bayley motor and development scores (7).

A cross-sectional study was designed to 190 children and evaluated delay followed by infant walker with 102 children in walker-user group and were reported that crawling, standing alone, and walking alone occurred later in this group significantly. They reported strong associations between the amount of using baby walker and extent of developmental delay and claimed that using infant walker may lead to delayed development. Despite these findings, the study has major methodological problems including failure to verify the outcome of the study and to randomize the study groups (15).

Age of gait acquisition was compared between children using baby walker and non-users and showed no statistically significant difference between two groups (376.17 ± 32.62 d and 378.75 ±27.99 d, respectively) (4). In this study, 26 infants were enrolled, among them 14 infants used baby walker, and gait acquisition was defined as ability to take five steps without any support (4). Finally, a recent study compared age at walking skills development and current motor development (using Alberta Infant Motor Scale) between two groups of walker users and non-users (10 subjects in each group) (16). They reported earlier age of walking skills development in walker user group compared to non-users (11.44 ± 1.87 months compared to 13.44 ± 2.00, respectively; *P*-value=0.044) while no difference was found between current motor development of two groups (*P-*value=0.566) (16). Besides, a case report in 1999 reported two cases that experienced disharmonic and delayed motor development, contractures of the calf-muscles and motor development mimicking spastic diplegia, and claimed that these symptoms occurred because of early use of baby walker (17). No more similar observations were reported later.

## Discussion

Although there are claims that using baby walker may lead to developmental delay in locomotor function (5, 14) and cognition (10), very small number of studies have evaluated these theories and approved them. Although pediatricians were aware of the risks and disadvantages of baby walker, 89% of them believed that there was lack of evidence on the subject (18). Evidence in the literature is not enough yet to prohibit parents from using baby walkers as well (10).

With respect to child development, two clinical trials (11, 12) were reviewed. Both of these trials showed no developmental delay followed by baby walker; however, they had major limitations that decrease the validity of their findings. Both of these trials were designed with very small number of subjects and they had defined the gait acquisition differently. 

Six observational studies were also reviewed. Among them, two cross-sectional studies (13, 15) and a retrospective cohort (7) had reported findings against baby walker and its negative effects on development. The study had a more powerful design compared to other observational studies (7). A relatively large sample was enrolled and evaluated child development through two different methods, clinical assessment and Bayley motor and developmental scores. A study with the largest sample size (190 children) reported strong associations between the amounts of baby walker use and extended of developmental delay (15). However, these findings are not reliable considering their major methodological problems. Delay in prone locomotion was reported among baby walker high users; however, authors found no delay in sitting or walking onset (13). Besides, their cross-sectional design had a small number of cases in each study group as limitation of their study.

Developmental delay using was evaluated DDST-S, which has a very different method compared to other studies. Although no delay among subjects was reported, findings could not be compared to similar studies. No developmental delay was reported in group (4), however, the number of patients was very small (26 subjects) in this study compared to other observational studies. The latest study in the issue was performed and had a qualitative design, using self-report questionnaires. Although they reported earlier age of walking skills development among walker users, their sample size is very small (20 subjects) and they have a problematic methodology. 

In comparison, studies with higher level of evidence do not approve any developmental delay because of baby walker, while larger observational studies with less methodological problems report the developmental delay. Data regarding negative role of walkers on child development is insufficient and conflicting, and a clear conclusion cannot be adapted. Use of baby walker must be with caution until conducting more studies powerful on the subject.

The main reasons for using baby walker among parents are as follows: providing enjoyment, facilitating child development, helping child to walk, safety of home environment, keeping the child quiet, encouraging mobility, providing exercise, and others (1, 2, 11). The main factor (in 79% of subjects) inhibiting mothers from using baby walker is the probability of accidents (19), while two third of infants with walker related injuries, continue using walkers (1). Parents do not believe baby walkers are dangerous (1). In addition, decision making on using of baby walker was not associated with awareness of its risks by parents (4). Baby walkers are not known as a dangerous equipment and even wrong beliefs exist about them (such as facilitating child development and helping child to walk) while we found no advantage for walkers regarding child development in previous studies.

In this study, we had some limitations: First, considering the different outcomes and definitions in reviewed articles and their reported results, we were not able to combine results and analyze them. Second, the study was limited to papers written in English. Third, we did not search all the available databases including Cochrane library. Fourth, injuries are highly associated with using baby walkers while we did not review papers on walker related injuries.

**In conclusion, **there is a huge lack of evidence on the possible effect of baby walker on child development. There is no evidence claiming that baby walkers can facilitate development or wake while few studies are present claiming the disadvantages of walkers on development. In addition, there is a gap on evaluation of the effect of walkers on cognitive development in previous studies. Current data available in the literature is not enough to prohibit using baby walker; however, it suggests no advantage of the walkers in child development. This issue must be noticed more by researchers to help parents decide better for their children, as well as pediatricians consulting their patients on this subject.

## Author's Contribution:

Omid Yaghini: conceptualized the study, helped in literature review, and approved the final manuscript as submitted. 

Shervin Badihian: carried out the literature review, drafted the manuscript, and approved the final manuscript as submitted.

 Negin Badihian: carried out the literature review, drafted the manuscript, and approved the final manuscript as submitted. 

All authors agreed to be accountable for all aspects of the work in ensuring that questions related to the accuracy or integrity of any part of the work are appropriately investigated and resolved.

Funding source**:** No funding was secured for this study

## Conflict of interest

The authors have no conflicts of interest to disclose.
